# Insulation in the Treatment of Rheumatism and Other Diseases

**Published:** 1874-05-15

**Authors:** P. M. Wagenhats

**Affiliations:** Lancaster, Ohio


### INSULATION IN THE TREATMENT OF RHEUMATISM AND OTHER DISEASES.

By P. M. Wagenhats, M.D., Lancaster, Ohio.

*From The Clinic.*

In the causation of disease we have closely established the relation of *heat* to the fluxes of the bowels. When *ozone* is in abundance in the atmosphere, we have learned to expect influenza and affections of the mucous membrane of the respiratory tract. We trace typhoid fever to the infection of potable water by sewerage; and we have seen scarlet fever follow the milkman's cart, and with the pabulum of life sow the seeds of death.

Rheumatism, asthma, and malarial fevers, have an acknowledged dependence upon the atmosphere as to temperature, — barometric pressure, and the presence of that tertium quid, vaguely called miasm. Electric tension has also its influence, which all, sick and well, recognize in their own persons. So, also, that material manifestation of force we know as *magnetism*, has, in all probability, a powerful influence in the causation of disease. Concerning this we have very few observations recorded. The field of research has rapidly enlarged as to its production of currents, etc., but it has never been studied as the *cause* of disease.

Whilst innumerable observations are on record as to its powers as a therapeutic agent, I have no theory to offer as to the *modus operandi* of the treatment to be detailed in the subsequent part of this article, but offer it as the truthful record of what is at present a limited experience, in hope that observations on this subject may be multiplied, and the facts lying in this direction may be studied.

On December 25, 1871, I was attacked with rheumatism of the ankle and the knee joints in one limb, then the other. I treated myself actively by alkalies, opiates, etc., in the ordinary manner recognized as of the most value in this disease. I was unable to leave my bed for three months, could not walk until April, 1872, and did not fully recover until the warm weather of June. On the 16th day of December, 1872, I was again assailed by my tormentor; treated myself as before, and "I thought myself happy" that I was able to be out of my room in eight weeks, privileged to hobble around the streets of our city with the aid of a cane. Warm weather again restored me to health, and during last summer and this winter I attended to my professional duties. On February 16, 1874, while congratulating myself that I should escape my annual attack, I was suddenly seized in the night-time with severe pains in both ankles. In the morning I failed, after an ardent effort, to leave my bed. Fever was intense, as also the swelling of ankle and knee joints. A sense of coldness of the lower extremities existed, which was even more distressing than the pain caused by the swelling of the joints. This condition continued until the morning of the 18th. From the 16th to 18th I was unable to sleep. On the morning of the 18th I *insulated* my bed, by causing the legs of the bedstead to be placed in four glass tumblers. In four hours thereafter the harrowing sense of coldness disappeared, yet the pain still continued. A sense of warmth and perspiration set in, and that night, at ten o'clock, I fell into a profound sleep, wakening in the morning of the 19th bathed in a profuse warm perspiration, without the aid of diaphoretics or anodynes.

I steadily improved, and in a few days was out of my room. On February 23d, I left home for Cincinnati, where I remained a week, during all of which time I felt neither pain or soreness in the articulations. I returned to my home on Saturday, and found next morning the disease returned. I at once insulated my bed, and in eight days was able to go to my office and engage in my professional duties.

George C——, age sixteen, during the autumn of 1873, had an attack of rheumatism, affecting nearly every joint in the body, and affecting the mitral valves of the heart. In the latter part of January, 1874, he was able to resume his labors. March 8th he had a relapse. I treated him in the usual way, without sensible improvement. I then concluded to try insulation, which I was slow to do in any case save my own, because it seemed whimsical. In six days after instituting insulation he left his bed, and was able to make a journey of eighteen miles, which he did without discomfort.

Mrs. ——, for eighteen years a sufferer from asthma, which occurred every month, without obvious connection with the menstrual function. During the cold months of the year she suffers almost continually. She had a severe attack in March, of this year, and in casting around for some *new* remedy, I concluded to insulate her bed. She was relieved in a short time, and since then, for more than a month, she has slept uninterruptedly upon the insulated bed, and has had no attack since.

I am aware that "one swallow does not make a summer;" and so small a number of observations does not establish the value of any form of treatment; yet, when I was so speedily relieved when I expected to follow the old course, I think there is value in it, and report these cases, promising another installment, for I have several under treatment.

